# Orphan nuclear receptor TLX contributes to androgen insensitivity in castration-resistant prostate cancer via its repression of androgen receptor transcription

**DOI:** 10.1038/s41388-018-0198-z

**Published:** 2018-03-20

**Authors:** Lin Jia, Dinglan Wu, Yuliang Wang, Wenxing You, Zhu Wang, Lijia Xiao, Ganhui Cai, Zhenyu Xu, Chang Zou, Fei Wang, Jeremy Yuen-Chun Teoh, Chi-Fai Ng, Shan Yu, Franky L. Chan

**Affiliations:** 1 School of Biomedical Sciences, Faculty of Medicine, The Chinese University of Hong Kong, Hong Kong, China; 2 School of Biomedical Sciences, Li Ka Shing Faculty of Medicine, The University of Hong Kong, Hong Kong, China; 3 Shenzhen Key Laboratory of Viral Oncology, The Clinical Innovation & Research Center (CIRC), Shenzhen Hospital, Southern Medical University, 518110 Shenzhen, China; 4 Department of Urology, The Hospital of Hainan Province, Haikou 570311 Hainan, China; 5 Department of Surgery, Faculty of Medicine, The Chinese University of Hong Kong, Hong Kong, China

## Abstract

The metastatic castration-resistant prostate cancer (CRPC) is a lethal form of prostate cancer, in which the expression of androgen receptor (AR) is highly heterogeneous. Indeed, lower AR expression and attenuated AR signature activity is shown in CRPC tissues, especially in the subset of neuroendocrine prostate cancer (NEPC) and prostate cancer stem-like cells (PCSCs). However, the significance of AR downregulation in androgen insensitivity and de-differentiation of tumor cells in CRPC is poorly understood and much neglected. Our previous study shows that the orphan nuclear receptor TLX (*NR2E1*), which is upregulated in prostate cancer, plays an oncogenic role in prostate carcinogenesis by suppressing oncogene-induced senescence. In the present study, we further established that TLX exhibited an increased expression in metastatic CRPC. Further analyses showed that overexpression of TLX could confer resistance to androgen deprivation and anti-androgen in androgen-dependent prostate cancer cells in vitro and in vivo, whereas knockdown of endogenous TLX could potentiate the sensitivity to androgen deprivation and anti-androgen in prostate cancer cells. Our study revealed that the TLX-induced resistance to androgen deprivation and anti-androgen was mediated through its direct suppression of *AR* gene transcription and signaling in both androgen-stimulated and -unstimulated prostate cancer cells. We also characterized that TLX could bind directly to *AR* promoter and repress *AR* transcription by recruitment of histone modifiers, including HDAC1, HDAC3, and LSD1. Together, our present study shows, for the first time, that TLX can contribute to androgen insensitivity in CRPC via repression of *AR* gene transcription and signaling, and also implicates that targeting the druggable TLX may have a potential therapeutic significance in CRPC management, particularly in NEPC and PCSCs.

## Introduction

The initial and advanced growth of prostate cancer is critically dependent on androgens. Hormone or androgen-deprivation therapy (ADT), which acts to suppress the androgen receptor (AR) signaling by androgen depletion or AR antagonists, is still the principal treatment option for locally advanced and metastatic prostate cancers. However, after initial favorable responses to hormone therapy, most patients inevitably develop resistance to treatment and relapse with a more aggressive form of castration-resistant prostate cancer (CRPC) within 2–3 years. Most studies on CRPC suggest that the advanced disease progression involves multiple and interrelating AR-dependent mechanisms, including (1) overexpression of AR and its co-regulators, (2) AR point mutations, (3) AR activation by growth factors-mediated signal transduction pathways, (4) AR-mediated activation of oncogenic fusion genes, (5) constitutively active AR splice variants, and (6) intratumoral production of androgens by either conversion from adrenal androgens or de novo androgen biosynthesis from cholesterol via overexpressed steroidogenic enzymes, that leading to persistent and reactivated AR signaling and altered androgen metabolism [1, 2]. Altered AR signaling is still the current research focus in CRPC.

AR signaling pathway is commonly altered and remains persistent in CRPC. Genomic amplification of *AR* gene has been found in 30% CPRC and even higher in 50% metastatic CRPC (mCRPC) [3–7]. However, the expression of AR is highly heterogeneous in prostate cancer specimens, reflecting the heterogeneity of the disease. Early immunohistochemical studies reveal that tumor tissues generally exhibit lower AR expression than normal and benign hyperplastic tissues, and also high Gleason score or less differentiated cancers display lower AR expression than low Gleason score cancers [8–13]. Quantitative real-time polymerase chain reaction (qRT-PCR) analysis on microdissected prostatic tissues confirms that tumor tissues express significant lower AR expression than normal and benign tissues [14]. Decreased AR expression is also shown in metastatic prostate cancer [12, 15]. A similar decrease or loss of AR immune reactivity is shown in CRPC and such loss of AR expression is demonstrated to be associated with poor prognosis and neuroendocrine differentiation (NED) in a subset of CRPC patients [11, 16–19]. Microarray gene expression analysis reveals that AR activity signature in prostate cancer tissues is decreased after hormone therapy and in CRPC [20]. However, the significance of downregulation of AR in advanced prostate cancer and CRPC progression is still poorly understood and neglected.

TLX (*NR2E1*) is an orphan nuclear receptor belonging to the nuclear receptor superfamily. Studies in past decades indicate that TLX plays important roles in brain and retina development, maintenance of stemness, and self-renewal of embryonic and adult neural stem cells (NSCs) via its direct transcriptional control of genes involved in DNA replication, cell cycle, adhesion, and migration, Wnt/α-catenin, and mitogen-activated protein kinase (MAPK) signaling [21, 22]. Its dysregulation is implicated in several neurological disorders [23]. Emerging evidences suggest that TLX performs oncogenic roles in oncogenesis and could be a potential therapeutic target for cancers [24]. Recent studies show that TLX plays an essential role in the development of brain tumors, as evidenced by its upregulation in high-grade gliomas and its transgenic overexpression can result in a long-term expansion of a subgroup of NSCs and form gliomas in mutant mouse brain in conjunction with genetic loss of tumor suppressors Pten or p53 [25–27]. TLX also performs a similar oncogenic role in neuroblastoma via its promotion of self-renewal of tumor-initiating cells and transactivation of *OCT4* gene [28]. Transgenic knockout and gene-knockdown studies suggest that in vivo targeting TLX is a potential therapeutic strategy for brain tumors [29, 30]. TLX is also shown to be upregulated in poor prognostic ERα-negative breast cancer [31]. Recently, we have demonstrated that TLX, which is upregulated in prostate cancer, can promote its initiation and progression by repression of oncogene-induced senescence via its differential co-regulation of senescence-regulatory genes *CDKN1A* and *SIRT1* [32].

In this study, we aim to elucidate the role of TLX in the androgen-insensitive growth of prostate cancer. Our study showed that TLX could directly repress the *AR* gene transcription via its direct binding and recruitment of chromatin modifiers as co-repressors to *AR* gene promoter. Our findings also implicate that TLX could be a potential therapy target for CRPC.

## Results

### TLX shows upregulation in CRPC xenograft model and human metastatic CRPC

**Fig. 1:**
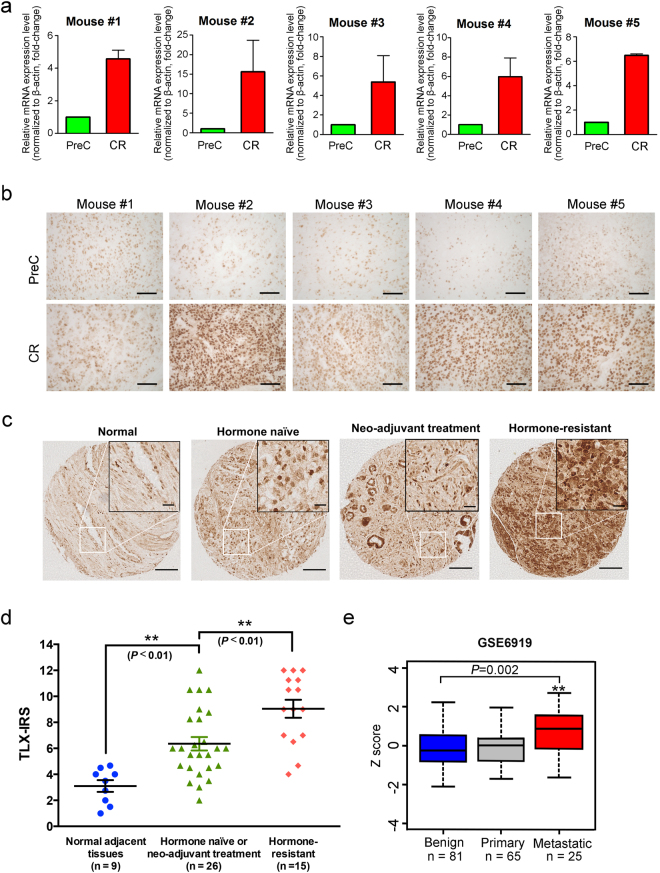
Increased expression of TLX in CRPC xenograft tumors and human metastatic prostate cancer tissues. **a**, **b** Real-time qRT-PCR and immunohistochemical analyses of TLX in VCaP-CRPC xenograft model. Significant increases of transcripts and immunosignals of TLX were detected in castration-relapse VCaP-CRPC xenograft tumors (CR) grown in castrated mice as compared to pre-castration (PreC) tumors grown in intact hosts. *n* = 7 per group. Data are presented as mean ± SD of triplicate measurements. Magnification, ×400; scale bars = 50 µm. **c** TLX immunohistochemistry. Representative micrographs of TLX-immunostained prostate cancer TMA. Magnification, ×40; bars, 250 µm. Insets show the enclosed areas at higher magnification. Magnification, ×400; bars, 30 µm. **d** TLX immunoreactive score analysis (TLX-IRS) performed on normal-adjacent tissues (*n* = 9), hormone-naïve or neo-adjuvant-treated prostate cancer (*n* = 26), and hormone-resistant tissues (*n* = 15). **P < 0.01 versus normal tissues. **e** Expression profile of TLX as revealed from a GEO dataset (GSE6919) [37]. TLX mRNA levels were compared in benign prostatic hyperplasia (BPH, *n* = 81), primary prostate cancer (*n* = 65), and metastatic hormone-refractory prostate cancer tissues (*n* = 25). Box plot (lines from top to bottom): maximum, third quartile Q3; median; minimum, first quartile Q1. **P < 0.002 versus BPH

We established CRPC a xenograft tumor model VCaP-CRPC, based on the castration-relapse growth of AR-positive and androgen-responsive VCaP cells in castrated host severe combined immunodeficiency (SCID) mice [33]. Expression analyses of TLX in VCaP-CRPC xenograft tumors revealed that both messenger RNA (mRNA) levels and immunosignals of TLX exhibited significant increased expression in castration-relapse VCaP-CRPC xenograft tumors grown in castrated mice as compared to pre-castration VCaP tumors grown in intact mice (Fig. 1a, b). qRT-PCR analysis of TLX expression in a panel of LNCaP- and VCaP-derived prostate cancer cell lines showed that TLX transcripts were significantly increased in androgen-independent LNCaP sublines (104R1, abl) established by long-term androgen deprivation [34, 35] and bicalutamide-resistant LNCaP-BC and VCaP-BC cells [36], as compared to their parental cells (Supplementary Figure S1a). To further validate the increased expression pattern of TLX in clinical CRPC, immunohistochemical analysis performed on a tissue microarray slide containing 56 validated cases of hormone-naïve and -refractory prostate cancer showed that TLX exhibited a significant increase in nuclear immunosignals in lesions in hormone-resistant tissues as compared to hormone-naive or neo-adjuvant-treated prostate cancer and normal prostatic tissues (Fig. 1c, d). Moreover, TLX expression was queried in publicly available gene expression databases and results showed that TLX exhibited a significant increased expression pattern in metastatic hormone-refractory prostate cancer as compared to benign prostatic hyperplasia and primary prostate cancer tissues from Gene Expression Omnibus dataset (GSE6919) [37] (Fig. 1e). To further explore the association of TLX and AR expression in primary prostate cancer and CRPC, we compared TLX levels in AR^low^ and AR^high^ subsets of prostate cancer patients using RNA-seq datasets in two published cohorts [19, 38] in an unbiased manner. Results showed that TLX exhibited upregulation in AR^low^, KLK3^low^, and KLK2^low^ subsets (Supplementary Figure S1b and S1c). These results suggest that increased TLX expression displays a positive association with the advanced progression of CRPC.

### TLX overexpression confers resistance to androgen deprivation and anti-androgen in AR-positive prostate cancer cells in vitro and in vivo

**Fig. 2:**
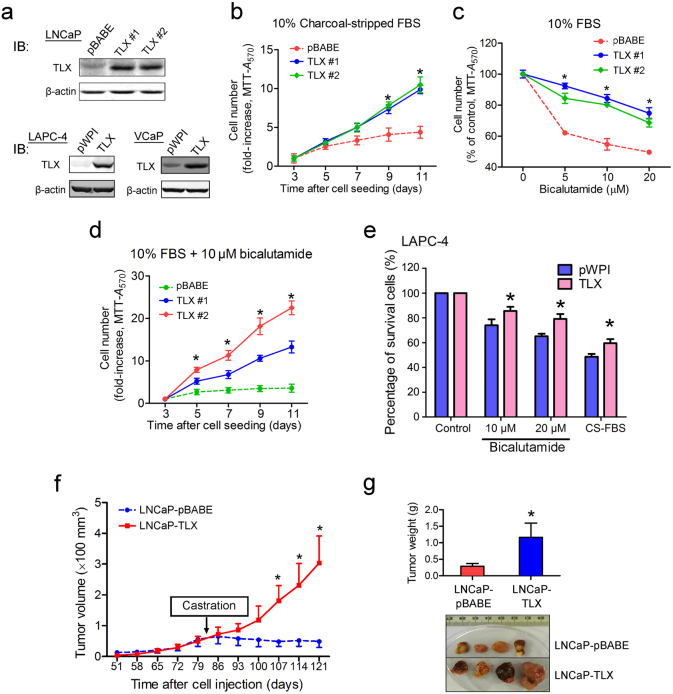
TLX overexpression enhances both in vitro and in vivo androgen deprivation-resistant growth capacity in AR-positive prostate cancer cells. **a** Immunoblot validation of stable TLX infectants generated from AR-positive LNCaP, LAPC-4 and VCaP cells. **b**–**d** In vitro growth responses of LNCaP-TLX infectants to charcoal-stripped fetal bovine serum (CS-FBS) and bicalutamide treatments. **e** In vitro growth responses of LAPC-4-TLX infectants to bicalutamide and CS-FBS. Data are presented as mean ± SD of triplicate assays. **f**, **g** In vivo tumorigenicity assay. **f** In vivo growth response of LNCaP-vector infectants and LNCaP-TLX xenograft tumors to host castration. Tumors formed by LNCaP-TLX infectants continued to grow aggressively in castrated hosts, whereas tumors formed by LNCaP-pBABE infectants ceased to grow upon castration. **g** Upper panel: graph shows the wet weights of dissected tumors formed by LNCaP-TLX and LNCaP-vector infectants, collected at 18 weeks after s.c. inoculation into intact host SCID mice, which were castrated at 9 weeks post inoculation. Lower panel: photograph shows the dissected tumors formed by the representative inoculated infectants. LNCaP-TLX infectants formed significantly larger tumors than LNCaP-vector infectants in castrated animals. *P < 0.05 versus LNCaP-vector infectants. Data are presented as mean ± SD of quadruplicate measurements

**Fig. 3:**
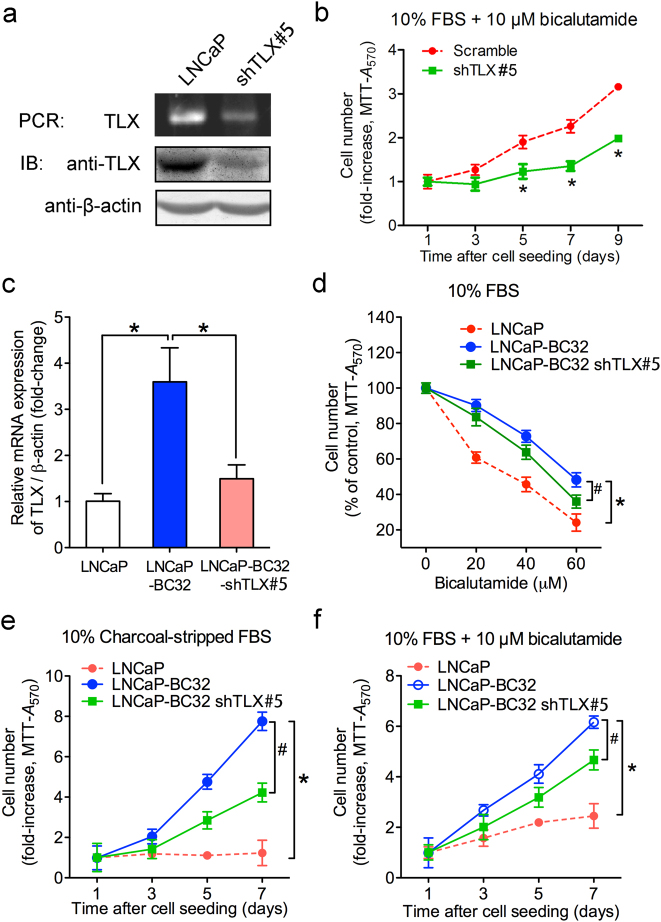
TLX knockdown potentiates sensitivity to bicalutamide and androgen deprivation in AR-positive prostate cancer cells. **a** RT-PCR and immunoblot validation of TLX knockdown in stable LNCaP-shTLX infectants. LNCaP-shTLX infectants showed reduced mRNA and protein expressions of TLX. **b** Growth response of LNCaP-shTLX and LNCaP-Scramble infectants to bicalutamide treatment by MTT assay. *P < 0.05 versus LNCaP-Scramble cells. Data are presented as mean ± SD of triplicate assays. **c** mRNA levels of TLX in bicalutamide-resistant LNCaP-BC32 cells and LNCaP-BC32-shTLX infectants as assayed by qRT-PCR. Data are shown as mean ± SD of triplicate assays. **d**–**f** In vitro growth responses of LNCaP-BC32 cells and LNCaP-BC32-shTLX infectants to bicalutamide (20–60 µM) and CS-FBS as assayed by MTT. *P < 0.05 versus LNCaP cells; ^#^*P* < 0.05 versus LNCaP-BC32 cells. Data are presented as mean ± SD of triplicate assays

Since TLX exhibited an increased expression in VCaP-CRPC xenograft model and mCRPC tissues, we hypothesize that TLX might perform a growth promoting role in CRPC. To elucidate the functional significance of TLX in prostate cancer growth upon androgen-deprivation stress, we then generated stable TLX-transduced clones in AR-positive prostate cancer cell lines (including LNCaP, VCaP, and LAPC-4) for in vitro and in vivo growth phenotype studies (Fig. 2a). In vitro growth characterization analyses showed that the immunoblot-validated TLX-overexpressing stable cells exhibited enhanced growth capacity (Supplementary Figure S2a and S2b) and higher resistant capacity to culture conditions with charcoal-stripped serum (CS; mimicking androgen deprivation) or bicalutamide and both (Fig. 2b–e), as compared to their counterpart empty vector infectants. In vivo tumorigenicity analysis showed that tumors formed by either LNCaP-TLX or their empty vector infectants grew at similar rates in intact SCID mice. Intriguingly, xenograft tumors formed by LNCaP-TLX-stable cells showed no response to castration of host mice and the tumors continued to grow aggressively after host castration, a sharp contrast to tumors formed by vector infectants that ceased to grow or become shrunk in castrated hosts (Fig. 2f, g). To further validate the significance of TLX in the induction of androgen-insensitive growth capacity in prostate cancer cells, we next evaluate the effects of knockdown of endogenous TLX expression in parental LNCaP cells and their bicalutamide-resistant LNCaP-BC cells (Fig. 3a, c), and examined their growth responses toward bicalutamide and CS treatments. In vitro growth analyses showed that short hairpin RNA (shRNA)-knockdown of TLX could inhibit the cell growth in LNCaP-shTLX cells (Supplementary Figure S2c) and potentiate their sensitivity to bicalutamide (Fig. 3b). LNCaP-BC32 cells showed more resistance to bicalutamide (20–60 µM) and 10% CS-FBS (Fig. 3d, e), and proliferated at faster rates upon bicalutamide (10 µM) treatment (Fig. 3f) as compared to their parental LNCaP cells. However, knockdown of TLX in LNCaP-BC32-shTLX cells restored the sensitivity to bicalutamide and CS-FBS treatments (Fig. 3d–f). These results showed that shRNA-knockdown of TLX could potentiate the sensitivity to bicalutamide and CS in both androgen-sensitive LNCaP cells and bicalutamide-resistant LNCaP-BC cells. Together, our results suggest that overexpression of TLX could promote androgen-deprivation- and anti-androgen-insensitive growth capacity in AR-positive prostate cancer cells.

### TLX acts to suppress AR expression and AR signaling, and upregulate neuroendocrine differentiation markers in prostate cancer cells

**Fig. 4:**
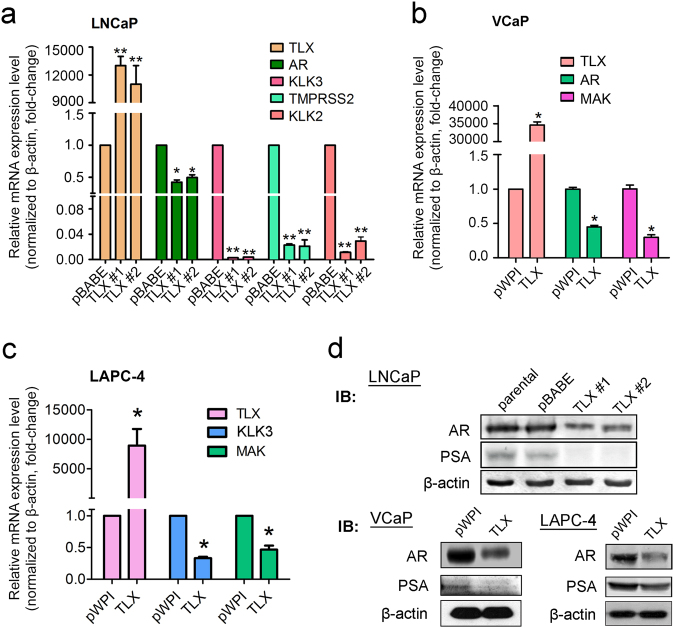
TLX overexpression induces downregulation of *AR* and its target genes in prostate cancer cells. **a** mRNA levels of *AR* and its targets (*KLK2*, *KLK3*, and *TMPRSS2*) in LNCaP-TLX infectants by qRT-PCR analysis. Data are presented as means ± SD of triplicate assays. **b**, **c** mRNA levels of *AR* and its targets (*KLK3* and *MAK*) in TLX-transduced VCaP and LAPC-4 cells by qRT-PCR analysis. *P < 0.05; **P < 0.01 versus empty vector-transduced cells. Data are presented as mean ± SD of triplicate assays. **d** Protein expressions of AR and PSA in TLX infectants of LNCaP, VCaP, and LAPC-4 cells by immunoblot analysis

We next sought to investigate whether the hormone-insensitive growth phenotype induced by ectopic TLX overexpression in prostate cancer cells could be associated with disturbance on AR signaling. Results of real-time qPCR showed that the mRNA levels of *AR* and AR-target genes (including *KLK3*/PSA, *KLK2*, *TMPRSS2*, and *MAK*) were significantly reduced in LNCaP, VCaP, and LAPC-4 TLX-overexpressing stable cells as compared to their vector-infectant counterparts (Fig. 4a–c). Results of immunoblots also confirmed that the protein levels of AR and PSA were significantly decreased in all three TLX-stable cells (Fig. 4d). Contrarily, mRNA and protein levels of AR and its targets showed significant upregulation in LNCaP-shTLX cells (Supplementary Figure S3c and S3d).

Since AR signaling is lost in neuroendocrine prostate cancer (NEPC), we next examined the possible association of TLX in NEPC progression in LNCaP-TLX cells. Expression analyses showed that the mRNA and protein levels of two NEPC markers ENO2 (neuronal-specific enolase) and CHGA (chromogranin A) exhibited significant higher expressions in LNCaP-TLX cells, but lower expressions in LNCaP-shTLX cells (Supplementary Figure S3). Moreover, the expressions of TLX and NEPC markers were also analyzed in AR^high^ and AR^low^ subsets of NEPC patients in Beltran NEPC cohort [19]. Results showed that the AR^low^ NEPC subset showed higher expressions of TLX and two critical NEPC-regulation factors *POU3F2* (*BRN2*) and *NCAM1* (Supplementary Figure S1c). These data suggest that TLX may play a role in the AR downregulation in NEPC and also its progression.

### TLX attenuates the androgen-stimulated transcriptional activity of AR in androgen-stimulated prostate cancer cells

**Fig. 5:**
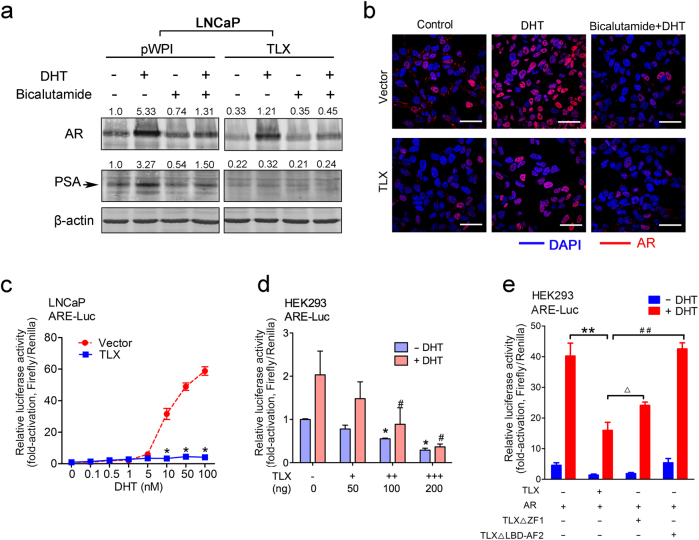
TLX suppresses androgen-stimulated transcriptional activity of AR in prostate cancer cells. **a** Immunoblot analysis of AR and PSA in LNCaP-TLX infectants upon treatments with AR agonist (dihydrotestosterone, DHT) and antagonist (bicalutamide). **b** Immunofluorescence analysis of AR in LNCaP-TLX infectants upon DHT and bicalutamide treatments. Magnification, ×600; scale bars = 50 µm. **c** Luciferase reporter assay of ARE-Luc performed in LNCaP-TLX infectants treated with serial dosages of DHT (0.1–100 nM). *P < 0.05 versus vector-infected LNCaP cells. Data are presented as mean ± SD of triplicate assays. **d** Luciferase reporter assay of ARE-Luc performed in DHT-treated or untreated non-prostatic HEK293 cells upon TLX transfection at increasing amount. *P < 0.05; ^#^*P* < 0.05 versus control vector-infected cells. Data are presented as mean ± SD of triplicate assays. **e** Luciferase reporter assay of ARE-Luc in HEK293 cells co-transfected with AR and TLX/TLX-mutants (TLXΔZF1 and TLXΔLBD-AF2) with or without DHT stimulation. ** and ^##^*P* < 0.01 versus co-transfection with TLX or TLXΔLBD-AF2; ^Δ^*P* < 0.05 versus TLXΔZF1. Data are presented as mean ± SD of triplicate assays

To further explore the molecular mechanism involved in the suppression of AR expression and its signaling in prostate cancer cells mediated by TLX overexpression, we next examined the basal and androgen-stimulated levels of the endogenous AR and PSA in LNCaP-TLX-stable cells. Immunoblot analysis showed that in the absence of dihydrotestosterone (DHT) or bicalutamide, LNCaP-TLX cells expressed lower levels of AR and PSA as compared to empty vector LNCaP-pPWI cells. DHT treatment induced significant increases of AR and PSA in LNCaP-pWPI cells, but only slight increase of AR and no change of PSA in LNCaP-TLX cells. Bicalutamide treatment, with or without DHT, attenuated expressions of both AR and PSA in LNCaP-pWPI cells, and further suppressed AR and PSA levels in LNCaP-TLX cells (Fig. 5a). These results suggested that the androgen-stimulated AR signaling pathway was interfered or blocked by TLX overexpression in prostate cancer cells. To further demonstrate the significance of TLX overexpression on the AR transactivation in androgen-stimulated prostate cancer cells, we examined the nuclear translocation of AR immunosignals in LNCaP-TLX infectants by immunofluorescence. Results showed that significant increase of AR immunosignals was induced in the nuclei of the LNCaP cells upon DHT treatment, but with such increase of immunosignals abolished by bicalutamide (Fig. 5b). Compared to vector-infected LNCaP cells, LNCaP-TLX infectants exhibited much weaker or undetected levels of AR immunosignals at their nuclei and cytoplasm; and upon DHT stimulation, LNCaP-TLX infectants expressed significant weaker nuclear immunosignals of AR and also with the immunosignals abolished by bicalutamide. The results suggest that TLX overexpression might interfere the AR transactivation. Notably, TLX did not exert any effects on the nuclear translocation of AR upon DHT stimulation, as most of the AR immunosignals were detected in nucleus rather than cytoplasm in both vector-infected and LNCaP-TLX infectants. Finally, we examined the significance of TLX on the transactivation of AR in LNCaP-TLX infectants by ARE-driven luciferase reporter assay. Results showed that LNCaP-TLX infectants exhibited no or negligible transactivation of ARE-luciferase reporter upon DHT treatment as compared to parental LNCaP cells, suggesting that overexpression of TLX could significantly suppress the AR transcription activity in prostate cancer cells (Fig. 5c). Moreover, TLX could also dose-dependently suppress the ARE-Luc reporter activity in DHT-treated or untreated HEK293 cells (Fig. 5d). However, the TLX-induced suppression on ARE was partially abolished by its DBD deletion (TLXΔZF1 with deletion of the first zinc finger at DBD) and completely rescued by LBD deletion (TLXΔLBD-AF2 with partial deletion of LBD), suggesting that intact DBD and LBD of TLX was necessary in TLX-mediated repression of AR transcription activity (Fig. 5e). Together, these results suggest that TLX may exert suppressive effect on the AR expression and its transcription activity in prostate cancer cells.

### TLX represses *AR* gene transcription via direct binding to its promoter

**Fig. 6:**
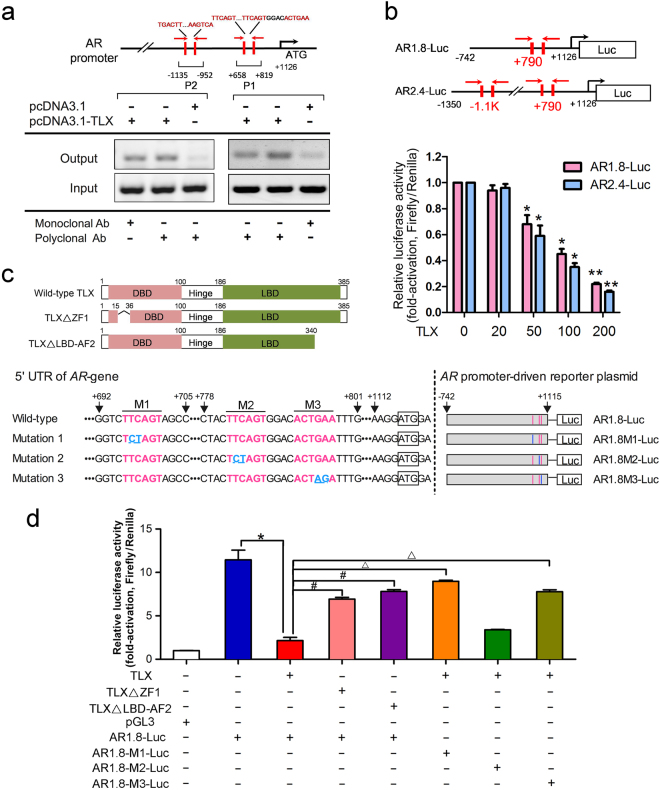
TLX-induced transrepression of *AR* is mediated by its direct binding to *AR* gene. **a** ChIP assay. Top: schematic diagram shows the locations of two identified TLX-binding sites (P1, P2) located at the 5′-UTR and proximal region of *AR* promoter. The TLX-binding motifs *TTCAGT* and palindromic sequence *ACTGAA* located at P1 and P2 sites are shown in red. P1 site contains more than one TLX-binding motifs. The locations of PCR primers (indicated by arrows) used for the ChIP assay are also shown. Bottom: ChIP assay of *AR* gene promoter performed in TLX-transfected or non-transfected HEK293 cells. Non-immunoprecipitated sonicated DNA (10% diluted) was used as input. **b** Top: schematic diagram shows the two *AR* gene promoter-driven constructs: pGL3-AR1.8-Luc and pGL3-AR2.4-Luc containing fragments of 742 bp and 1350 bp of the *AR* gene promoter, respectively, and the full-length of the 5′-UTR downstream to the start codon *ATG* (+1126). AR1.8-Luc contains the P1 site and AR2.4-Luc contains both the P1 and P2 sites (highlighted in red). Bottom: luciferase reporter assay of *AR* gene promoter (AR1.8-Luc and AR2.4-Luc) transactivation performed in HEK293 cells transfected with TLX or empty vector pcDNA3.1. **P < 0.01 versus empty vector. Data are presented as mean ± SD of triplicate assays. **c** Top: schematic diagrams show the structural organization of the wild-type intact TLX (385 aa) and truncated mutants TLXΔZF1 (364 aa; with deletion of first zinc finger in DBD) and TLXΔLBD-AF2 (340 aa; with deletion of AF2 in LBD). Bottom left: schematic diagrams show the wild-type sequences of the two putative TLX-binding motifs *TTCAGT* (M1 and M2) and one palindromic sequence *ACTGAA* (M3) (highlighted in red) located at the P1 site of the 5′-UTR of *AR* gene, the point mutations at M1-M3 TLX-binding motifs (highlighted in blue) introduced by site-directed mutagenesis, and also the generated reporter constructs AR1.8-Luc carrying the wild-type P1 and AR1.8M1/M2/M3-Luc carrying the point mutations at P1 site. **d** Luciferase reporter assay of wild-type and site-mutated *AR* promoter-driven reporters (AR1.8M1-Luc, AR1.8M2-Luc, and AR1.8M3-Luc) performed in HEK293 cells transfected with TLX or its truncated mutants. *^,#,Δ^*P* < 0.05. Data are presented as mean ± SD of triplicate assays

We next sought to determine whether the TLX-induced downregulation of AR expression in prostate cancer cells could be mediated by its direct transrepression of *AR* gene expression or indirectly via other TLX-controlled downstream signaling pathways. Chromatin immunoprecipitation (ChIP) analysis performed in TLX-transfected HEK293 cells identified two TLX-binding sites, located at 819–658 bp (designated as P1) at the 5′-UTR and −1135 to −952 bp (P2) upstream of the *ATG* start site (Fig. 6a). In order to demonstrate the significance of the identified TLX-binding sites on their repression on *AR* gene induced by TLX, two *AR* promoter-driven luciferase reporter constructs were used for reporter gene assay: AR1.8-Luc containing the P1 site and AR2.4 containing both P1 and P2 sites. Results of luciferase reporter assays performed in HEK293 cells showed that transfected TLX could significantly suppress the activities of both AR1.8-Luc and AR2.4-Luc reporters in a dose-dependent manner, with more suppression on AR2.4-Luc (Fig. 6b). These results suggest that TLX exerts its transrepression regulation on *AR* gene via multiple sites. Sequence analysis revealed two potential TLX-binding motifs *TTCAGT* and one palindromic sequence *ACTGAA* located at the P1 site of the 5′-UTR of *AR* (Fig. 6c). Based on these results, we then generated three AR1.8-Luc constructs (AR1.8M1/M2/M3-Luc) with point mutations introduced by site-directed mutagenesis at three identified potential TLX-binding motifs (Fig. 6c), in order to provide further insight into how TLX would bind to these motifs at the 5′-UTR of *AR*. Results of luciferase reporter assay showed that the AR1.8-Luc activity was significantly repressed in HEK293 cells when transfected with intact TLX but much less with truncated mutants (TLXΔZF1 and TLXΔLBD-AF2), suggesting that maximum repression of *AR* gene expression would require TLX with intact DBD and LBD (Fig. 6d). Our results also showed that the reporter activity was much less repressed in cells transfected with AR1.8M1-Luc and AR1.8M3-Luc reporters carrying point mutations at M1 and M3 sites, but showing similar repression magnitude as AR1.8-Luc with wild-type sequence (Fig. 6d). These results further suggest that TLX prefers binding to the M1 and M3 sites of the 5′-UTR of the *AR* gene as a homodimer to mediate repression of *AR*, in a similar manner as it binds to the *CDKN1A* (p21^WAF1/CIP1^) gene promoter as shown previously [32]. Together, these results suggest that TLX could repress the *AR* gene transcription via its direct binding to consensus TLX-binding sequences present at the 5′-UTR and promoter of *AR* gene.

### TLX-mediated repression of *AR* gene transcription involves recruitment of chromatin modifiers as co-repressors

**Fig. 7:**
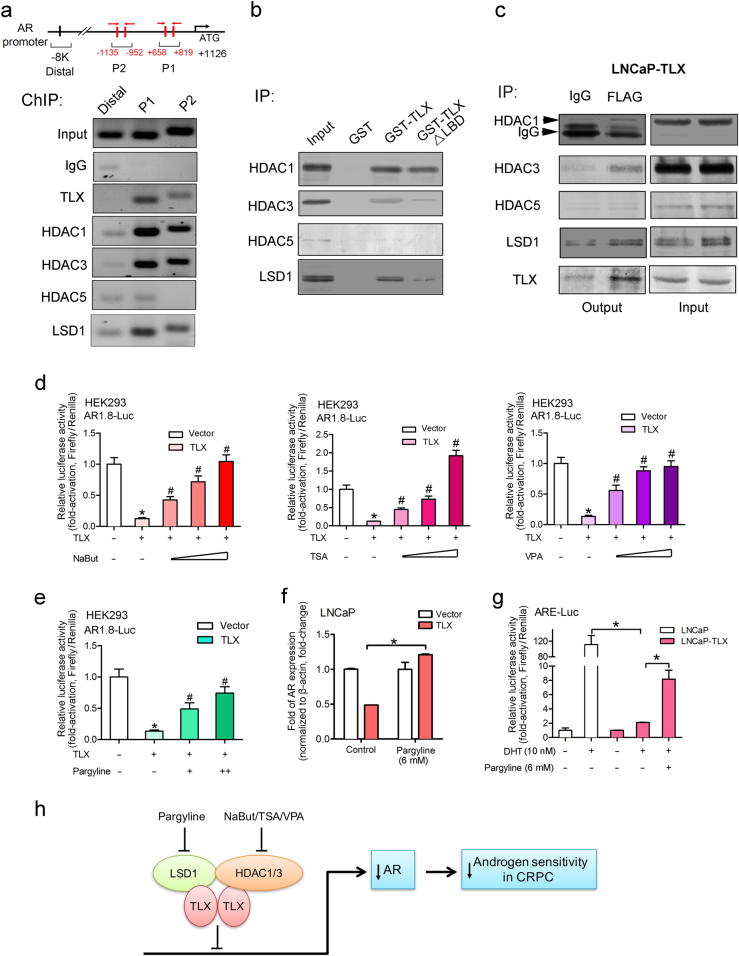
TLX recruits HDACs and LSD1 as co-repressors to suppress the *AR* gene expression. **a** ChIP assay of *AR* gene promoter performed in LNCaP cells infected with pWPI-TLX. Top: schematic diagram shows the locations of the identified TLX-binding sites P1 and P2 located at the 5′-UTR and proximal region of *AR* gene promoter, and also the locations of PCR primers used for ChIP assay. Sonicated DNA fragments extracted from LNCaP-TLX infectants were immunoprecipitated with various antibodies (against TLX, HDACs, and LSD1) and IgG as antibody specificity control. PCR of *AR* 5′-UTR and promoter region was performed using primers flanking the TLX-binding motifs at P1 and P2 sites, and also the 8-kb upstream distal region as negative control. DNA fragments flanking the P1 and P2 sites could be intensely PCR-amplified from extracted DNA immunoprecipitated by TLX, HDAC1, HDAC3, and LSD1, but barely or negatively by HDAC5 and IgG. **b** GST pull-down assay performed in LNCaP-TLX infectants. Cellular extracts from LNCaP-TLX infectants were incubated with GST-TLX or GST-TLXΔLBD fusion proteins, and the pull-down proteins were probed by antibodies against HDAC1, HDAC3, HDAC5, and LSD1. **c** Immunoprecipitation (IP) assay performed in FLAG-TLX-infected LNCaP cells. Cell lysates were immunoprecipitated with a FLAG-antibody or IgG as IP control and immunoblot-analyzed with HDACs and LSD1 antibodies. **d** Luciferase reporter assay of AR1.8-Luc performed in TLX-transfected HEK293 cells treated or untreated with pan-inhibitors of HDACs, NaBut (0.5–2.0 mM), TSA (0.25–1.0 µg/ml), and VPA (2–8 mM). *P < 0.05 versus empty vector; ^#^*P* < 0.05 versus untreated TLX-transfected cells. Data are presented as mean ± SD of triplicate assays. **e** Luciferase reporter assay of AR1.8-Luc in TLX-transfected HEK293 cells treated with LSD1 inhibitor pargyline (3–6 mM). *P < 0.05 versus empty vector; ^#^*P* < 0.05 versus untreated TLX-transfected cells. Data are presented as mean ± SD of triplicate assays. **f** qRT-PCR analysis of AR expression in LNCaP-TLX infectants treated with pargyline (6 mM). *P < 0.05 versus untreated cells. Data are presented as mean ± SD of triplicate assays. **g** Luciferase reporter assay of ARE-Luc in LNCaP-TLX infectants treated or untreated with pargyline and DHT. *P < 0.05 versus DHT-stimulated and pargyline-untreated cells. **h** Schematic diagram depicts the hypothesized mechanism of TLX-mediated repression of *AR* transcriptional activity via its recruitment of HDACs and LSD1 as co-repressors and its significance in induction of androgen insensitivity in CRPC progression

It is previously shown that TLX can perform transrepressive regulation of its target genes via a mechanism of recruitment of chromatin modifiers as co-repressors, including histone deacetylases (HDACs) and lysine-specific demethylase (LSD1) [39, 40]. In order to determine whether HDACs and LSD1 would be recruited by TLX to the identified TLX-enriched sites at the *AR* gene, we performed the ChIP assay in LNCaP cells infected with lentiviral vector pWPI-TLX. Sonicated chromatin fragments were immunoprecipitated with antibodies against FLAG, HDACs, and LSD1. Results of ChIP-PCR assay showed that DNA fragments flanking either the P1 or P2 sites could be immunoprecipitated by HDAC1, HDAC3, and LSD1 antibodies but not HDAC5, and PCR-amplified (Fig. 7a). In vitro glutathione *S*-transferase (GST) pull-down assay was then performed to further validate whether TLX could directly interact with HDACs and LSD1 so as to execute its transrepression function. Results of immunoblot analysis showed that HDAC1, HDAC3, and LSD1 could be immunodetected in the GST-TLX protein complex, but these chromatin modifiers were much less accumulated in the GST-TLXΔLBD complex, indicating that interaction between TLX and chromatin modifiers would require TLX with intact LBD (Fig. 7b). To validate whether the endogenous TLX could interact with chromatin modifiers, IP assay was performed in FLAG-TLX-infected LNCaP cells. Results showed that HDAC1, HDAC3, and LSD1 were associated with TLX, whereas association with HDAC5 was not detected (Fig. 7c). Together, these results indicate that TLX recruits HDAC1, HDAC3, and LSD1 to the *AR* gene and repress *AR* transcription. To further validate the significance of the identified TLX-associated chromatin modifiers on their contribution to the repression of *AR* gene transcription, we then performed the luciferase reporter assays on TLX-transfected non-prostatic HEK293 cells treated with pan-inhibitors of HDACs (including NaBut, TSA, and VPA) and LSD1 (pargyline). Results showed that treatments with HDAC and LSD1 inhibitors could dose-dependently reverse and restore the repressed AR-Luc reporter activity mediated by the transfected TLX in HEK293 cells (Fig. 7d, e). Moreover, pargyline treatment could also significantly restore the repressed AR expression in LNCaP-TLX infectants and its transcription in androgen-stimulated LNCaP-TLX infectants (Fig. 7f, g). Together, these results suggest that TLX recruits both HDACs and LSD1 as co-repressors and together the TLX-HDACs-LSD1 complex contributes to the transrepression regulation of *AR* gene transcription.

## Discussion

The current view on the persistent and reactivated AR signaling in CRPC, that forms the basis of targeting the AR-axis signaling in ADT, is largely based on the facts of positive AR expression detected in clinical CRPC tumor tissues [11, 41], persistent AR activity and intraprostatic androgen levels in primary prostate tumors from androgen deprivation-treated patients [42], recurrent serum PSA levels and also the persistent AR activity as shown in experimental xenograft models of CRPC [43–47]. On the other hand, the prognostic significance of AR expression in the progression of prostate cancer and CRPC still remains controversial [48]. Besides, AR expression in metastatic and non-metastatic CRPC is still not established largely due to limited access of clinical specimens. Some reports demonstrate that lower AR expression and attenuated AR signature activity is indeed shown in clinical CRPC tissues [11, 17, 20] and also mCRPC [12, 15, 49, 50]. Contrarily, rebound of AR expression is demonstrated in some experimental CRPC xenograft models upon short-term castration [43, 45]. Prospective analysis suggests that the decrease or loss of AR expression in CRPC may be associated with its advanced progression to androgen-independent NED progression and poor prognosis [17, 51]. AR is reported to be suppressed by N-Myc in NEPC [51] and the p38MAPK/FOXC2/Zeb1 pathway in prostate cancer stem-like cells (PCSCs), and inhibition of FOXC2 can reduce stem cell properties and restore AR/PSA expression and also sensitivity to docetaxel in DU145 cells [52]. These new evidences suggest that downregulation of AR may exert an oncogenic role in advanced CRPC, at least some subpopulations of CRPC; and restoration of AR expression and its signaling might be beneficial to some advanced patients with NEPC or mCRPC patients with cancer stem-like cell phenotype. However, how the decrease or loss of AR is regulated in CRPC progression and through which CRPC acquires androgen insensitivity, NED, and stemness phenotypes, are still poorly understood.

In the present study, we demonstrated that the orphan nuclear receptor TLX, which displayed an upregulation expression in both clinical mCRPC and xenograft models, could confer androgen insensitivity in AR-positive prostate cancer cells in vitro and in vivo. Our functional analyses further revealed that the TLX-induced androgen insensitivity was mediated through its direct repression of *AR* gene transcription and suppression of AR transcriptional activity in prostate cancer cells via a mechanism of recruitment of chromatin modifiers HDACs and LSD1 to the TLX-binding motifs at the *AR* gene promoter. We also observed that the expressions of at least two NED markers (ENO2 and CHGA) were significantly upregulated in TLX infectants. Our newly published results show that TLX exhibits an upregulation in PCSCs enriched by three-dimensional cultures [33]. Our previous study also showed that TLX can play a role in suppression of oncogene-induced senescence in prostate cancer cells via its direct repression of p21/*CDKN1A* and *SIRT1* [32]. Therefore, we hypothesize that TLX may contribute to NEPC acquisition by its suppression of AR and promote NEPC and PCSCs proliferation at least partially by its differential transcriptional regulation of *CDKN1A* and *SIRT1*. However, the specific role of TLX in NEPC progression and stemness of advanced prostate cancer need further investigation.

Prostate cancer is a heterogeneous disease and exhibits different clinical behaviors in patients. Indeed, subsets of PSA^−/low^/PSA^high^ and AR^low^/AR^high^ populations can be distinguished among prostate cancer patients [53, 54]. Our in vitro results showed that TLX overexpression could suppress significantly AR expression and its targets PSA/KLK3 and KLK2 in LNCaP-TLX cells (Fig. 4a); whereas treatment with DHT could only trigger the AR expression but not PSA in LNCaP-TLX cells (Fig. 5a). Previously, we show in an anti-androgen-resistant LNCaP-BC model that the AR transcription activity is significantly repressed in LNCaP-BC cells [36], which express high TLX levels. It remains to be determined that if TLX could suppress AR signaling by suppression of AR transcriptional activity in the bulk of the differentiated AR^high^/PSA^high^ cell populations in castration-relapse CRPC tumors. Our present findings show that TLX can directly target and repress the *AR* gene promoter, and also suppress the ligand-activated transcriptional activity of AR in prostate cancer cells, and through which it could enhance the NED progression and stemness of PCSCs in subpopulations of CRPC. We also showed that histone modifiers HDACs and LSD1, whose overexrepression are implicated in CRPC progression [55, 56], can function as co-repressors of TLX and participate in TLX-mediated repression of *AR* transcription. Indeed, TLX can interact with multiple co-regulators to mediate its transcriptional activity in target cells [57]. However, the underlying mechanism still needs further characterization.

In summary, our current study provides evidences showing that the orphan nuclear receptor TLX can promote the androgen insensitivity in CRPC progression by repressing *AR* transcription via recruitment of histone modifiers HDACs and LSD1 (Fig. 7h). Our findings also implicate that targeting TLX signaling could be a potential therapeutic strategy for CRPC treatment.

## Materials and methods

### Chemicals and antibodies

5α-Androstan-17β-ol-3-one (4,5α-dihydrotestosterone, DHT), bicalutamide, pargyline hydrochloride, sodium butyrate (NaBut), trichostatin A (TSA), and valproic acid (VPA) were purchased from Sigma-Aldrich (St. Louis, MO, USA). The rabbit polyclonal TLX-antibody was immunoblot-validated as described previously [32]. Other antibodies used in this study are listed as follows: monoclonal anti-TLX was purchased from Perseus Proteomics Inc. (Tokyo, Japan); rabbit polyclonal antibodies anti-histone deacetylase 1 (HDAC1), -HDAC3, -HDAC5, and mouse monoclonal anti-β-actin from Cell Signaling Technology (Danvers, MA, USA); anti-LSD1 from abcam (Hong Kong, China); anti-AR and anti-PSA from Santa Cruz Biotechnology (Dallas, TX, USA).

### Cell cultures

Human prostate cancer cell lines (including LNCaP, VCaP, and LAPC-4) and their sublines (including androgen-insensitive sublines LNCaP-abl, LNCaP-104-R1; bicalutamide-resistant sublines LNCaP-BC32, LNCaP-BC56, VCaP-BC36, and VCaP-BC62) were used in this study. LNCaP, LAPC-4, HEK293, and PA317 cell lines were obtained from ATCC (Manassa, Virginia, USA). VCaP cells were kindly provided by Dr. Kenneth Pienta (University of Michigan); LNCaP-104-R1 cells from Dr. Shutsung Liao (University of Chicago); LNCaP-abl cells from Dr. Helmet Klocker (University of Innsbruck). LNCaP-abl cells were maintained in RPMI1640 supplemented with 10% CS-FBS and LNCaP-104R1 cells were cultured in DMEM with 10% CS-FBS. LNCaP-BC and VCaP-BC cells were established as described previously [36]. All cell lines were authenticated by STR profiling and tested for mycoplasma contamination before use.

### Human prostatic tissues and immunohistochemistry

Human prostate cancer tissue microarray was provided by Department of Defense Prostate Cancer Research Program, Prostate Cancer Biorepository Network (PCBN), NYU. Peroxidase immunohistochemistry was performed on paraffin sections of formaldehyde-fixed prostatic tissues as described previously [58].

### Plasmid construction

(a) Expression plasmids pBABE-TLX, pWPI-TLX, pcDNA3.1-TLX, and truncated mutant pcDNA3.1-TLXΔZF1 and pcDNA3.1-TLXΔLBD-AF2 were generated as described previously [32]. Full-length TLX and deletion mutant TLXΔLBD were subcloned into pGEX as pGEX-TLX and pGEX-TLXΔLBD for GST-fusion protein production in *Escherichia coli*. (b) Reporter plasmids: ARE-Luc was kindly provided by Dr. J.M. Vanacker (Lyon, France) [59]. pGL3-AR1.8 and pGL3-AR2.4 were gifts

from Dr. Angelo Poletti [60]. pGL3-AR1.8M1, -AR1.8M2, and -AR1.8M3 were generated by sequential site-directed mutagenesis of TLX-binding sites *AAGTCA* to *AGATCA* [61].

### Transduction and generation of stable TLX-overexpressed clones

The production of retrovirus and lentivirus, and generation of stable TLX-overexpressed cells, AR-positive prostate cancer cells (including LNCaP, VCaP, and LAPC-4) was performed as described previously [62, 63].

### RNA interference

Procedures on the generation of stable TLX-knockdown prostate cancer cells by shRNA were described previously [32].

### In vitro cell growth assay

In vitro cell growth of TLX- or shTLX-transduced prostate cancer cells were evaluated by MTT assay as described previously [62].

### Immunofluorescence microscopy

Procedures on AR immunofluorescence were described previously [64].

### PCR and immunoblot analyses

Detailed procedures on PCR and immunoblotting are described as previously [62] and primers for RT-PCR were listed in Supplementary Table 1.

### In vivo tumorigenicity assay and VCaP-CRPC model

LNCaP-TLX and vector-control cells were subcutaneously inoculated into the flank region of intact male SCID mice and allowed to grow for 11 weeks when tumors reached sizes of 1 cm^3^ or larger. Host mice were then orchidectomized and allowed to grow for another 6–7 weeks. Tumor sizes were measured weekly. The VCaP-CRPC xenograft model was established previously [33]. All animal protocols were approved by the Animal Experimentation Ethics Committee, Chinese University of Hong Kong.

### Luciferase reporter assays

For ARE-Luc and AR-Luc reporter assay performed in HEK 293 cells, plasmids were transduced into cells by Lipofectamine 2000 (Life Technologies, California, US). For ARE-Luc reporter assay performed in LNCaP and LNCaP-TLX cells, cells were transduced with pGL3-ARE-Luc and pRL-SV40 by electroporation with an optimized exponential pulse of 230 V and 950 μF (Gene Pulser Xcell Electroporation System, Bio-Rad). After 24 h post transfection, cells were treated with hormone (DHT) or epigenetic modifiers (TSA, VPA, NaBut, or pargyline) or vehicles (DMSO, ethanol, or water) as control, and incubated for another 24 h. Cells were lysed for luciferase luminescence measurement using the *Renilla*-firefly dual-luciferase assay as described previously [65].

### Chromatin immunoprecipitation

ChIP assay on *AR* gene promoter was performed in HEK293 cells transfected with pcDNA3.1-TLX/pcDNA3.1 or TLX-transduced LNCaP cells, following procedure as described previously [65]. Genomic DNA extracted from TLX-transfected or -transduced cells was sonicated and sheared into fragments. DNA samples were immunoprecipitated by various antibodies (against TLX, HDACs, and LSD1), followed by PCR analysis (30 cycles) with primer pairs specific for human *AR* gene promoter (P1: 5′-CGGAGCCAGAGATCAAAAGA-3′; 5′-CAAAATCCTCCACCTTCCAA-3′; P2: 5′-AAGAGACCCAGGCAAAAATC-3′; 5′-GTGGAGATGCAAGTGGGAAT-3′; distal primers: 5′-GTGCATGCTGTGCTTCACT-3′; 5′-GTGCCTTCACCCTGTCTT-3′).

### Immunoprecipitation assay

Procedures on co-immunoprecipitation (Co-IP) assay were performed as previously descripted [66].

### GST pull-down assay

BL2 (DE3) competent *E. coli* cells were transformed with pGEX-TLX or pGEX-TLXΔLBD plasmids to express the GST-fusion recombinant proteins GST-TLX_1–385_ and GST-TLXΔLBD_1–340_. After protein induction with isopropyl-β-d-thiogalactopyranoside (IPTG), bacterial cells were sonicated in TIF lysis buffer (150 mM NaCl, 20 mM Tris, 1 mM MgCl_2_, 0.1% NP40, 10% glycerol, pH 8.0) and the GST-fusions were affinity-purified with glutathione-sepharose 4B resin (GE Healthcare). For pull-down of overexpressed TLX, cell lysates of pWPI-TLX- and pWPI-TLXΔLBD-transduced LNCaP cells were incubated with the GST-fusion protein-bound beads overnight with rotation at 4 °C. After washing the beads with TIF buffer, the protein complexes were eluted with Laemmli-SDS sample buffer, separated by SDS-PAGE, and visualized by immunoblotting.

### Statistical analysis

All results were expressed as mean ± SD. Statistical analysis of data was performed using two-tailed Student’s *t* test and differences were considered significant where *P* < 0.05.

## Electronic supplementary material

Supplementary figure S1-S3 and figure legends(PDF 808 kb)

Supplementrary Table 1(DOCX 12 kb)

## References

[1] Ferraldeschi R, Welti J, Luo J, Attard G, de Bono JS (2015). Targeting the androgen receptor pathway in castration-resistant prostate cancer: progresses and prospects. Oncogene.

[2] Katsogiannou M, Ziouziou H, Karaki S, Andrieu C, Henry de Villeneuve M, Rocchi P (2015). The hallmarks of castration-resistant prostate cancers. Cancer Treat Rev.

[3] Visakorpi T, Hyytinen E, Koivisto P, Tanner M, Keinanen R, Palmberg C (1995). In vivo amplification of the androgen receptor gene and progression of human prostate cancer. Nat Genet.

[4] Palmberg C, Koivisto P, Hyytinen E, Isola J, Visakorpi T, Kallioniemi OP (1997). Androgen receptor gene amplification in a recurrent prostate cancer after monotherapy with the nonsteroidal potent antiandrogen Casodex (bicalutamide) with a subsequent favorable response to maximal androgen blockade. Eur Urol.

[5] Koivisto P, Kononen J, Palmberg C, Tammela T, Hyytinen E, Isola J (1997). Androgen receptor gene amplification: a possible molecular mechanism for androgen deprivation therapy failure in prostate cancer. Cancer Res.

[6] Linja MJ, Savinainen KJ, Saramaki OR, Tammela TL, Vessella RL, Visakorpi T (2001). Amplification and overexpression of androgen receptor gene in hormone-refractory prostate cancer. Cancer Res.

[7] Brown RS, Edwards J, Dogan A, Payne H, Harland SJ, Bartlett JM (2002). Amplification of the androgen receptor gene in bone metastases from hormone-refractory prostate cancer. J Pathol.

[8] Masai M, Sumiya H, Akimoto S, Yatani R, Chang CS, Liao SS (1990). Immunohistochemical study of androgen receptor in benign hyperplastic and cancerous human prostates. Prostate.

[9] Segawa N, Mori I, Utsunomiya H, Nakamura M, Nakamura Y, Shan L (2001). Prognostic significance of neuroendocrine differentiation, proliferation activity and androgen receptor expression in prostate cancer. Pathol Int.

[10] Chodak GW, Kranc DM, Puy LA, Takeda H, Johnson K, Chang C (1992). Nuclear localization of androgen receptor in heterogeneous samples of normal, hyperplastic and neoplastic human prostate. J Urol.

[11] Ruizeveld de Winter JA, Janssen PJ, Sleddens HM, Verleun-Mooijman MC, Trapman J, Brinkmann AO (1994). Androgen receptor status in localized and locally progressive hormone refractory human prostate cancer. Am J Pathol.

[12] Li R, Wheeler T, Dai H, Frolov A, Thompson T, Ayala G (2004). High level of androgen receptor is associated with aggressive clinicopathologic features and decreased biochemical recurrence-free survival in prostate: cancer patients treated with radical prostatectomy. Am J Surg Pathol.

[13] Takeda H, Akakura K, Masai M, Akimoto S, Yatani R, Shimazaki J (1996). Androgen receptor content of prostate carcinoma cells estimated by immunohistochemistry is related to prognosis of patients with stage D2 prostate carcinoma. Cancer.

[14] Rosner IL, Ravindranath L, Furusato B, Chen Y, Gao C, Cullen J (2007). Higher tumor to benign ratio of the androgen receptor mRNA expression associates with prostate cancer progression after radical prostatectomy. Urology.

[15] Fleischmann A, Rocha C, Schobinger S, Seiler R, Wiese B, Thalmann GN (2011). Androgen receptors are differentially expressed in Gleason patterns of prostate cancer and down-regulated in matched lymph node metastases. Prostate.

[16] Pertschuk LP, Schaeffer H, Feldman JG, Macchia RJ, Kim YD, Eisenberg K (1995). Immunostaining for prostate cancer androgen receptor in paraffin identifies a subset of men with a poor prognosis. Lab Invest.

[17] Komiya A, Yasuda K, Watanabe A, Fujiuchi Y, Tsuzuki T, Fuse H (2013). The prognostic significance of loss of the androgen receptor and neuroendocrine differentiation in prostate biopsy specimens among castration-resistant prostate cancer patients. Mol Clin Oncol.

[18] Beltran H, Rickman DS, Park K, Chae SS, Sboner A, MacDonald TY (2011). Molecular characterization of neuroendocrine prostate cancer and identification of new drug targets. Cancer Discov.

[19] Beltran H, Prandi D, Mosquera JM, Benelli M, Puca L, Cyrta J (2016). Divergent clonal evolution of castration-resistant neuroendocrine prostate cancer. Nat Med.

[20] Mendiratta P, Mostaghel E, Guinney J, Tewari AK, Porrello A, Barry WT (2009). Genomic strategy for targeting therapy in castration-resistant prostate cancer. J Clin Oncol.

[21] Islam MM, Zhang CL (2015). TLX: a master regulator for neural stem cell maintenance and neurogenesis. Biochim Biophys Acta.

[22] Wang T, Xiong JQ (2016). The orphan nuclear receptor TLX/NR2E1 in neural stem cells and diseases. Neurosci Bull.

[23] O'Leary J. D., O'Leary O. F., Cryan J. F., Nolan Y. M. (2016). Regulation of behaviour by the nuclear receptor TLX. Genes, Brain and Behavior.

[24] Wu D, Cheung A, Wang Y, Yu S, Chan FL (2016). The emerging roles of orphan nuclear receptors in prostate cancer. Biochim Biophys Acta.

[25] Liu HK, Wang Y, Belz T, Bock D, Takacs A, Radlwimmer B (2010). The nuclear receptor tailless induces long-term neural stem cell expansion and brain tumor initiation. Genes Dev.

[26] Park HJ, Kim JK, Jeon HM, Oh SY, Kim SH, Nam DH (2010). The neural stem cell fate determinant TLX promotes tumorigenesis and genesis of cells resembling glioma stem cells. Mol Cells.

[27] Zou Y, Niu W, Qin S, Downes M, Burns DK, Zhang CL (2012). The nuclear receptor TLX is required for gliomagenesis within the adult neurogenic niche. Mol Cell Biol.

[28] Chavali PL, Saini RK, Zhai Q, Vizlin-Hodzic D, Venkatabalasubramanian S, Hayashi A (2014). TLX activates MMP-2, promotes self-renewal of tumor spheres in neuroblastoma and correlates with poor patient survival. Cell Death Dis.

[29] Zhu Z, Khan MA, Weiler M, Blaes J, Jestaedt L, Geibert M (2014). Targeting self-renewal in high-grade brain tumors leads to loss of brain tumor stem cells and prolonged survival. Cell Stem Cell.

[30] Cui Q, Yang S, Ye P, Tian E, Sun G, Zhou J (2016). Downregulation of TLX induces TET3 expression and inhibits glioblastoma stem cell self-renewal and tumorigenesis. Nat Commun.

[31] Lin ML, Patel H, Remenyi J, Banerji CR, Lai CF, Periyasamy M (2015). Expression profiling of nuclear receptors in breast cancer identifies TLX as a mediator of growth and invasion in triple-negative breast cancer. Oncotarget.

[32] Wu D, Yu S, Jia L, Zou C, Xu Z, Xiao L (2015). Orphan nuclear receptor TLX functions as a potent suppressor of oncogene-induced senescence in prostate cancer via its transcriptional co-regulation of the *CDKN1A* (p21(WAF1) (/) (CIP1)) and *SIRT1* genes. J Pathol.

[33] Wang Z, Wu D, Ng CF, Teoh JY, Yu S, Wang Y (2018). Nuclear receptor profiling in prostatospheroids and castration-resistant prostate cancer. Endocr Relat Cancer.

[34] Kokontis J, Takakura K, Hay N, Liao S (1994). Increased androgen receptor activity and altered c-myc expression in prostate cancer cells after long-term androgen deprivation. Cancer Res.

[35] Culig Z, Hoffmann J, Erdel M, Eder IE, Hobisch A, Hittmair A (1999). Switch from antagonist to agonist of the androgen receptor bicalutamide is associated with prostate tumour progression in a new model system. Br J Cancer.

[36] Yu S, Jia L, Zhang Y, Wu D, Xu Z, Ng CF (2013). Increased expression of activated endothelial nitric oxide synthase contributes to antiandrogen resistance in prostate cancer cells by suppressing androgen receptor transactivation. Cancer Lett.

[37] Chandran UR, Ma C, Dhir R, Bisceglia M, Lyons-Weiler M, Liang W (2007). Gene expression profiles of prostate cancer reveal involvement of multiple molecular pathways in the metastatic process. BMC Cancer.

[38] Taylor BS, Schultz N, Hieronymus H, Gopalan A, Xiao Y, Carver BS (2010). Integrative genomic profiling of human prostate cancer. Cancer Cell.

[39] Sun G, Yu RT, Evans RM, Shi Y (2007). Orphan nuclear receptor TLX recruits histone deacetylases to repress transcription and regulate neural stem cell proliferation. Proc Natl Acad Sci USA.

[40] Yokoyama A, Takezawa S, Schule R, Kitagawa H, Kato S (2008). Transrepressive function of TLX requires the histone demethylase LSD1. Mol Cell Biol.

[41] van der Kwast TH, Schalken J, Ruizeveld de Winter JA, van Vroonhoven CC, Mulder E, Boersma W (1991). Androgen receptors in endocrine-therapy-resistant human prostate cancer. Int J Cancer.

[42] Mostaghel EA, Page ST, Lin DW, Fazli L, Coleman IM, True LD (2007). Intraprostatic androgens and androgen-regulated gene expression persist after testosterone suppression: therapeutic implications for castration-resistant prostate cancer. Cancer Res.

[43] Chen CD, Welsbie DS, Tran C, Baek SH, Chen R, Vessella R (2004). Molecular determinants of resistance to antiandrogen therapy. Nat Med.

[44] Locke JA, Guns ES, Lubik AA, Adomat HH, Hendy SC, Wood CA (2008). Androgen levels increase by intratumoral de novo steroidogenesis during progression of castration-resistant prostate cancer. Cancer Res.

[45] Cai C, Wang H, Xu Y, Chen S, Balk SP (2009). Reactivation of androgen receptor-regulated TMPRSS2:ERG gene expression in castration-resistant prostate cancer. Cancer Res.

[46] Cai C, He HH, Chen S, Coleman I, Wang H, Fang Z (2011). Androgen receptor gene expression in prostate cancer is directly suppressed by the androgen receptor through recruitment of lysine-specific demethylase 1. Cancer Cell.

[47] Knuuttila M, Yatkin E, Kallio J, Savolainen S, Laajala TD, Aittokallio T (2014). Castration induces up-regulation of intratumoral androgen biosynthesis and androgen receptor expression in an orthotopic VCaP human prostate cancer xenograft model. Am J Pathol.

[48] Tamburrino L, Salvianti F, Marchiani S, Pinzani P, Nesi G, Serni S (2012). Androgen receptor (AR) expression in prostate cancer and progression of the tumor: lessons from cell lines, animal models and human specimens. Steroids.

[49] Davis JN, Wojno KJ, Daignault S, Hofer MD, Kuefer R, Rubin MA (2006). Elevated E2F1 inhibits transcription of the androgen receptor in metastatic hormone-resistant prostate cancer. Cancer Res.

[50] Tomlins SA, Mehra R, Rhodes DR, Cao X, Wang L, Dhanasekaran SM (2007). Integrative molecular concept modeling of prostate cancer progression. Nat Genet.

[51] Dardenne E, Beltran H, Benelli M, Gayvert K, Berger A, Puca L (2016). N-Myc induces an EZH2-mediated transcriptional program driving neuroendocrine prostate cancer. Cancer Cell.

[52] Paranjape AN, Soundararajan R, Werden SJ, Joseph R, Taube JH, Liu H (2016). Inhibition of FOXC2 restores epithelial phenotype and drug sensitivity in prostate cancer cells with stem-cell properties. Oncogene.

[53] Qin J, Liu X, Laffin B, Chen X, Choy G, Jeter CR (2012). The PSA(-/lo) prostate cancer cell population harbors self-renewing long-term tumor-propagating cells that resist castration. Cell Stem Cell.

[54] Wise D, Armenia J, Chen Y, Nelson P, Schultz N, Sawyers CL (2017). The immunomodulatory protein Dickkopf-1 (DKK1) defines a non-neuroendocrine subtype of metastatic castration-resistant prostate cancer (mCRPC) with low AR and low PSA expression. J Clin Oncol.

[55] Weichert W, Roske A, Gekeler V, Beckers T, Stephan C, Jung K (2008). Histone deacetylases 1, 2 and 3 are highly expressed in prostate cancer and HDAC2 expression is associated with shorter PSA relapse time after radical prostatectomy. Br J Cancer.

[56] Kahl P, Gullotti L, Heukamp LC, Wolf S, Friedrichs N, Vorreuther R (2006). Androgen receptor coactivators lysine-specific histone demethylase 1 and four and a half LIM domain protein 2 predict risk of prostate cancer recurrence. Cancer Res.

[57] Corso-Diaz X, de Leeuw CN, Alonso V, Melchers D, Wong BK, Houtman R (2016). Co-activator candidate interactions for orphan nuclear receptor NR2E1. BMC Genomics.

[58] Cheung CP, Yu S, Wong KB, Chan LW, Lai FM, Wang X (2005). Expression and functional study of estrogen receptor-related receptors in human prostatic cells and tissues. J Clin Endocrinol Metab.

[59] Teyssier C, Bianco S, Lanvin O, Vanacker JM (2008). The orphan receptor ERRalpha interferes with steroid signaling. Nucleic Acids Res.

[60] Vismara G, Simonini F, Onesto E, Bignamini M, Miceli V, Martini L (2009). Androgens inhibit androgen receptor promoter activation in motor neurons. Neurobiol Dis.

[61] Zhao C, Sun G, Li S, Shi Y (2009). A feedback regulatory loop involving microRNA-9 and nuclear receptor TLX in neural stem cell fate determination. Nat Struct Mol Biol.

[62] Yu S, Wang X, Ng CF, Chen S, Chan FL (2007). ERRgamma suppresses cell proliferation and tumor growth of androgen-sensitive and androgen-insensitive prostate cancer cells and its implication as a therapeutic target for prostate cancer. Cancer Res.

[63] Yu S, Wang MW, Yao X, Chan FL (2009). Establishment of a novel immortalized human prostatic epithelial cell line stably expressing androgen receptor and its application for the functional screening of androgen receptor modulators. Biochem Biophys Res Commun.

[64] Cai G, Wu D, Wang Z, Xu Z, Wong KB, Ng CF (2017). Collapsin response mediator protein-1 (CRMP1) acts as an invasion and metastasis suppressor of prostate cancer via its suppression of epithelial-mesenchymal transition and remodeling of actin cytoskeleton organization. Oncogene.

[65] Yu S, Wong YC, Wang XH, Ling MT, Ng CF, Chen S (2008). Orphan nuclear receptor estrogen-related receptor-beta suppresses in vitro and in vivo growth of prostate cancer cells viap21(WAF1/CIP1) induction and as a potential therapeutic target in prostate cancer. Oncogene.

[66] Zou C, Yu S, Xu Z, Wu D, Ng CF, Yao X (2014). ERRalpha augments HIF-1 signalling by directly interacting with HIF-1alpha in normoxic and hypoxic prostate cancer cells. J Pathol.

