# Supplementary material for: Orphan nuclear receptor TLX contributes to androgen insensitivity in castration-resistant prostate cancer via its repression of androgen receptor transcription
Source: Oncogene. 2018 Mar 20;37(25):3340–55. doi: 10.1038/s41388-018-0198-z (PMC6013422; doi:10.1038/s41388-018-0198-z)
Supplement: Supplementary file 1 — Supplementary figure S1-S3 and figure legends(PDF 808 kb) [file 41388_2018_198_MOESM1_ESM.pdf]

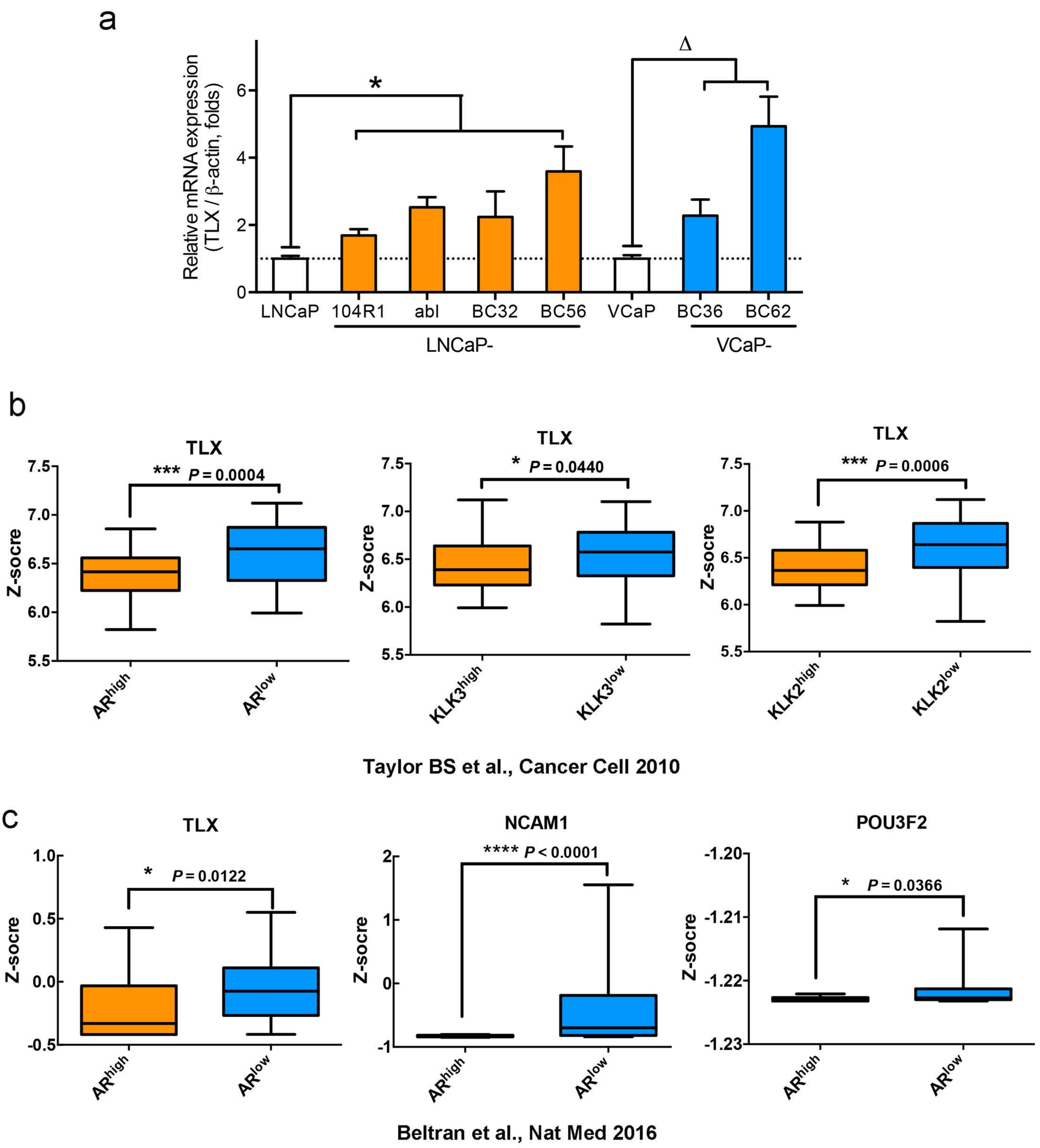

Supplementary Figure S1

Supplementary Figure S1. (**a**) Increased expression of TLX in androgen-insensitive LNCaP sublines (abl, 104R1) and bicalutamide-resistant sublines of LNCaP (BC32, BC56) and VCaP (BC36, BC62) prostate cancer cells as analyzed by qRT-PCR. Data are presented as mean ± SD obtained from three independent real-time qPCR experiments. *, $P < 0.05$ versus parental LNCaP or VCaP cells. (**b**) Expression of TLX in $AR^{high/low}$, $KLK3^{high/low}$ and $KLK2^{high/low}$ population subsets (high and low are defined by their Z-scores > or < median values: 9.53 for AR, 13.08 for KLK3, 12.86 for KLK2) of primary prostate tumors as revealed in MSKCC Cancer Cell 2010 RNA-seq dataset downloaded via the cBioPortal Cancer Genomics website (http://wwwcbioportal.org/). TLX exhibits higher expression levels in AR^{low}, $KLK3^{low}$ and KLK2 ^{low} subsets of primary prostate cancer. (**c**) Expression patterns of TLX and two critical NEPC-regulation factors *POU3F2* and *NCAM1* in AR^{high} and AR^{low} subsets of CRPC as revealed in Trent/Cornell/Broad 2016 RNA-seq dataset. TLX and AR manifest a significant negative expression correlation in $AR^{high/low}$ subsets of CRPC (high and low are defined here by AR Z-score > or < median value 15.91).

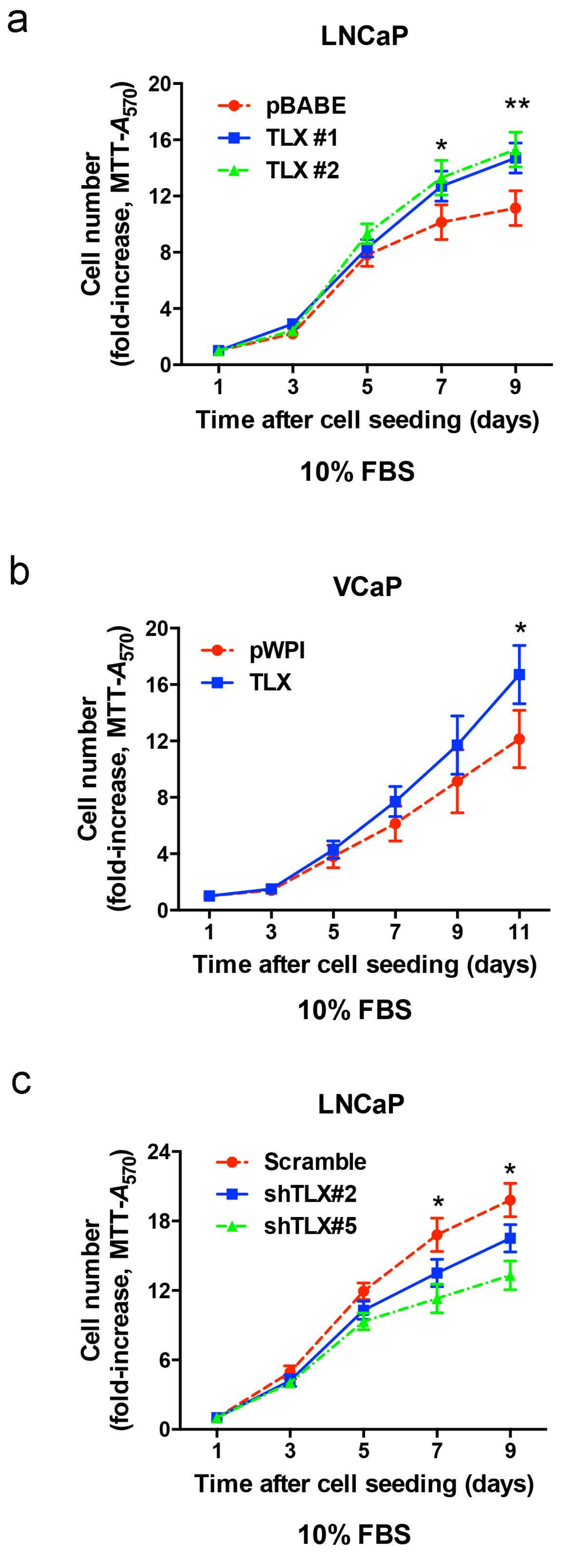

Supplementary Figure S2

Supplementary Figure S2. TLX promotes prostate cancer cell proliferation. (**a** and **b**) *In vitro* growth of TLX-overexpression infectants of LNCaP and VCaP cells as assayed by cell counting. Ectopic TLX overexpression could significantly promote prostate cancer cell growth. *, $P < 0.05$, **, $P < 0.01$ versus vector control. (**c**) *In vitro* growth of LNCaP-shTLX infectants by cell counting. Knockdown of endogenous TLX could suppress the growth of LNCaP cells. *, $P < 0.05$ versus scramble control. Data are presented as mean ± SD of triplicate assays.

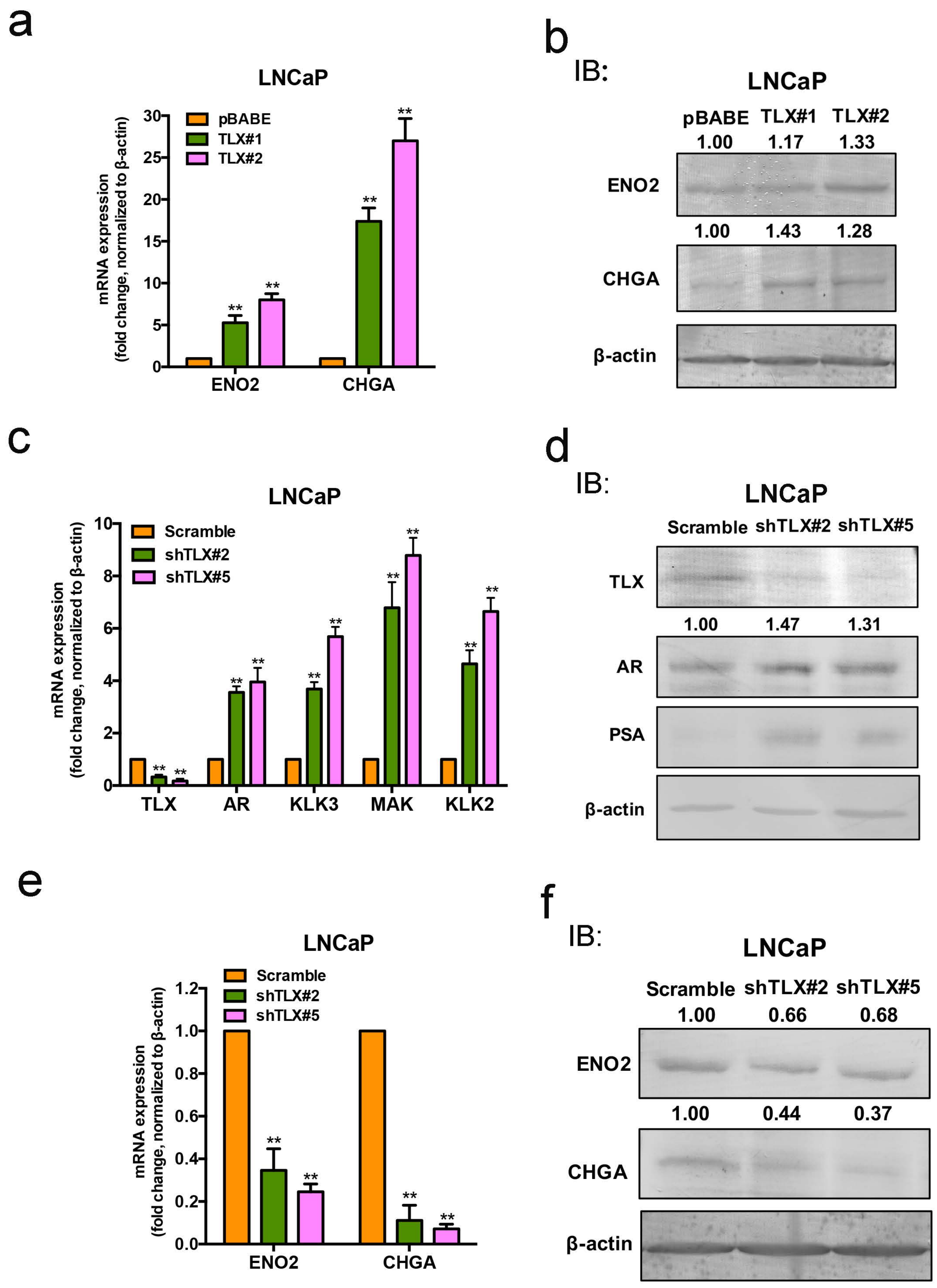

Supplementary Figure S3

Supplementary Figure S3. TLX upregulates the expression of NED markers. (**a** and **b**) LNCaP-shTLX stable infectants. Knockdown of endogenous TLX could increase mRNA and protein expressions of AR and its targets (*KLK3/PSA*, *MAK* and *KLK2*) in LNCaP-shTLX cells. (**c** and **d**) LNCaP-TLX stable infectants. Ectopic overexpression of TLX could enhance mRNA and protein expressions of two NED markers ENO2 and CHGA in LNCaP-TLX cells. (**e** and **f**) LNCaP-shTLX stable infectants. Knockdown of endogenous TLX suppressed both mRNA and protein expressions of ENO2 and CHGA in LNCaP-shTLX cells. **, $P < 0.01$ versus vector control. Data are presented as mean ± SD of triplicate assays.
