# Supplementary material for: Orphan nuclear receptor TLX contributes to androgen insensitivity in castration-resistant prostate cancer via its repression of androgen receptor transcription
Source: Oncogene. 2018 Mar 20;37(25):3340–55. doi: 10.1038/s41388-018-0198-z (PMC6013422; doi:10.1038/s41388-018-0198-z)
Supplement: Supplementary file 2 — Supplementrary Table 1(DOCX 12 kb) [file 41388_2018_198_MOESM2_ESM.docx]

| Genes | Forward primer (5’-3’) | Reverse primer (5’-3’) |
| --- | --- | --- |
| *TLX (NR2E1)* | CAAACGGAGCATCCGAAGGAA | AAACACTTCTTCAGCCGACAC |
| *AR* | CGGAAGCTGAAGAAACTTGG | ATGGCTTCCAGGACATTCAG |
| *TMPRSS2* | CTCTCCCTAACCCCTTGTCC | AGAGGTGACAGCTCCATGCT |
| *KLK2* | TACCACCCTGGGGTTATGAA | GCCCCACCGTCTTGTTACTA |
| *KLK3 (*PSA*)* | TTGTCTTCCTCACCCTGTCC | TCACGCTTTTGTTCCTGATG |
| *MAK* | GGTCACAGCCACCATACACTGA | ACTTCCAACAGCCCACACATC |
| *ACTB (*β-actin*)* | ATGGATGATGATATCGCCGCG | CTCCATGTCGTCCCAGTTGGT |

**Supplementary Table 1**

Primer pairs used for SYBR Green-based RT-PCR:
